# Tobacco Cessation Interventions and Smoke-Free Policies in Mental Health and Substance Abuse Treatment Facilities — United States, 2016

**DOI:** 10.15585/mmwr.mm6718a3

**Published:** 2018-05-11

**Authors:** Kristy Marynak, Brenna VanFrank, Sonia Tetlow, Margaret Mahoney, Elyse Phillips, Ahmed Jamal, MBBS, Anna Schecter, Doug Tipperman, Stephen Babb

**Affiliations:** ^1^Office on Smoking and Health, National Center for Chronic Disease Prevention and Health Promotion, CDC; ^2^Office of Policy, Planning, and Innovation, Substance Abuse and Mental Health Services Administration.

Persons with mental or substance use disorders or both are more than twice as likely to smoke cigarettes as persons without such disorders and are more likely to die from smoking-related illness than from their behavioral health conditions (*1*,*2*). However, many persons with behavioral health conditions want to and are able to quit smoking, although they might require more intensive treatment (*2*,*3*). Smoking cessation reduces smoking-related disease risk and could improve mental health and drug and alcohol recovery outcomes (*1*,*3*,*4*). To assess tobacco-related policies and practices in mental health and substance abuse treatment facilities (i.e., behavioral health treatment facilities) in the United States (including Puerto Rico), CDC and the Substance Abuse and Mental Health Services Administration (SAMHSA) analyzed data from the 2016 National Mental Health Services Survey (N-MHSS) and the 2016 National Survey of Substance Abuse Treatment Services (N-SSATS). In 2016, among mental health treatment facilities, 48.9% reported screening patients for tobacco use, 37.6% offered tobacco cessation counseling, 25.2% offered nicotine replacement therapy (NRT), 21.5% offered non-nicotine tobacco cessation medications, and 48.6% prohibited smoking in all indoor and outdoor locations (i.e., smoke-free campus). In 2016, among substance abuse treatment facilities, 64.0% reported screening patients for tobacco use, 47.4% offered tobacco cessation counseling, 26.2% offered NRT, 20.3% offered non-nicotine tobacco cessation medications, and 34.5% had smoke-free campuses. Full integration of tobacco cessation interventions into behavioral health treatment, coupled with implementation of tobacco-free campus policies in behavioral health treatment settings, could decrease tobacco use and tobacco-related disease and could improve behavioral health outcomes among persons with mental and substance use disorders (*1*–*4*).

SAMHSA conducts N-MHSS and N-SSATS annually among all known public and private facilities in the United States that provide mental health or substance abuse treatment services.* Survey respondents are typically facility administrators or others knowledgeable about facility operations; web-based and paper options for completion are available. In 2016, 12,745 eligible mental health treatment facilities responded to N-MHSS (response rate = 91.1%) and 14,632 eligible substance abuse treatment facilities responded to N-SSATS (91.4%). Facilities in U.S. territories, except Puerto Rico, and facilities that did not respond to one or more tobacco-related questions assessed in this report were excluded, yielding a total of 12,136 mental health and 14,263 substance abuse treatment facilities.^†^ Descriptive statistics were assessed nationally and by state.

In 2016, tobacco screening was the most commonly implemented tobacco-related practice in mental health (48.9%) and substance abuse (64.0%) treatment facilities (Table). Cessation counseling was the most commonly offered tobacco dependence treatment in mental health (37.6%) and substance abuse (47.4%) treatment facilities. Approximately one fourth of all mental health (25.2%) and substance abuse (26.2%) treatment facilities offered NRT, and approximately one fifth of mental health (21.5%) and substance abuse (20.3%) treatment facilities offered non-nicotine medications. Approximately half of mental health (48.6%) and one third of substance abuse treatment facilities (34.5%) reported having smoke-free campuses. Among facilities with smoke-free campuses, 57.3% of mental health and 39.4% of substance abuse treatment facilities did not report offering counseling, 67.6% of mental health and 65.7% of substance abuse treatment facilities did not report offering NRT, and 74.6% and 75.8% did not report offering non-nicotine medications.

**TABLE:** Number and percentage of mental health and substance abuse treatment facilities that offer tobacco screening and cessation treatment and that prohibit smoking in all indoor and outdoor settings, by type of facility — National Mental Health Services Survey and National Survey of Substance Abuse Treatment Services, United States, including Puerto Rico, 2016

Characteristic/Location	Mental health treatment facilities*						Substance abuse treatment facilities^†^					
	No. of facilities	% Offering treatment/smoke-free campus					No. of facilities	% Offering treatment/smoke-free campus				
		Screening for tobacco use	Smoking/Tobacco cessation counseling	Nicotine replacement therapy	Non-nicotine cessation medications	Smoke-free campus		Screening for tobacco use	Smoking/Tobacco cessation counseling	Nicotine replacement therapy	Non-nicotine cessation medications	Smoke-free campus
**Overall^§^**	**12,136**	**48.9**	**37.6**	**25.2**	**21.5**	**48.6**	**14,263**	**64.0**	**47.4**	**26.2**	**20.3**	**34.5**
**Facility type**												
Private for-profit	2,152	41.6	36.3	24.0	19.7	39.2	5,044	54.9	39.1	19.3	16.3	22.4
Private nonprofit	7,700	47.0	34.1	21.1	17.9	52.8	7,600	67.5	50.5	28.0	20.2	41.3
Public agency/department	2,284	61.9	50.6	40.1	35.0	43.3	1,619	75.7	58.7	39.5	32.9	40.7
**State**												
Alabama	193	39.9	31.1	26.4	19.2	31.1	135	34.8	37.0	17.0	10.4	10.4
Alaska	99	57.6	38.4	22.2	15.2	67.7	94	78.7	54.3	17.0	13.8	47.9
Arizona	377	46.7	38.7	17.2	21.0	27.3	355	62.0	43.1	30.1	27.3	30.1
Arkansas	235	32.8	27.2	16.6	11.5	41.3	113	51.3	48.7	20.4	12.4	47.8
California	877	37.6	26.9	17.2	13.1	41.2	1,413	51.5	42.3	19.6	15.6	22.4
Colorado	185	55.7	48.6	32.4	25.4	61.1	393	63.6	45.8	19.8	17.8	34.1
Connecticut	230	52.6	44.8	33.0	32.2	57.8	223	79.4	55.6	43.5	35.4	39.0
Delaware	29	41.4	37.9	20.7	24.1	55.2	45	60.0	40.0	26.7	20.0	33.3
District of Columbia	41	46.3	36.6	14.6	17.1	51.2	34	38.2	32.4	23.5	17.6	32.4
Florida	488	47.1	35.2	26.8	19.1	45.7	706	55.4	44.8	33.4	22.8	28.9
Georgia	219	42.9	27.4	20.1	16.4	39.3	311	45.7	32.8	19.9	16.7	25.1
Hawaii	45	48.9	62.2	33.3	40.0	42.2	174	82.8	66.7	6.3	5.2	65.5
Idaho	176	24.4	20.5	10.8	13.6	19.9	140	42.1	30.0	10.7	15.0	10.0
Illinois	391	42.5	30.7	24.8	20.5	43.5	671	50.1	28.2	16.5	13.1	24.6
Indiana	301	67.8	56.8	37.5	35.9	73.8	262	69.1	48.1	26.3	26.0	59.5
Iowa	155	38.7	26.5	20.0	16.8	58.1	163	78.5	43.6	29.4	18.4	58.9
Kansas	119	35.3	21.8	21.8	14.3	44.5	200	41.0	33.5	14.5	14.0	22.5
Kentucky	221	41.2	22.6	16.7	11.8	34.8	361	57.1	26.9	13.9	9.1	15.8
Louisiana	186	54.8	44.1	37.1	31.7	43.5	150	65.3	49.3	40.7	24.7	30.7
Maine	203	49.8	36.0	11.8	11.8	59.1	228	72.4	49.1	21.1	16.2	46.5
Maryland	291	45.0	34.4	19.2	17.2	45.4	397	71.8	49.4	20.7	13.4	30.5
Massachusetts	337	50.1	39.5	27.6	21.4	57.3	351	87.2	77.5	43.9	35.3	34.2
Michigan	359	49.0	41.5	28.4	22.8	49.0	477	56.2	38.8	19.3	15.3	32.3
Minnesota	240	52.9	39.6	26.7	25.8	44.6	369	58.3	31.2	24.1	16.5	15.2
Mississippi	180	39.4	30.6	21.1	16.7	38.9	94	43.6	37.2	26.6	16.0	25.5
Missouri	219	59.4	50.7	42.9	32.9	55.3	286	61.9	44.1	24.5	19.9	28.3
Montana	88	42.0	25.0	17.0	17.0	39.8	64	50.0	39.1	29.7	17.2	26.6
Nebraska	128	54.7	32.0	22.7	18.8	43.0	136	61.0	41.2	26.5	24.3	35.3
Nevada	51	39.2	27.5	23.5	15.7	23.5	80	56.3	46.3	31.3	27.5	40.0
New Hampshire	61	67.2	50.8	41.0	32.8	55.7	64	78.1	59.4	34.4	34.4	37.5
New Jersey	318	37.7	37.4	23.6	20.8	42.5	368	67.7	54.1	24.2	16.3	29.1
New Mexico	72	44.4	34.7	34.7	19.4	48.6	153	60.8	34.6	22.2	20.9	34.0
New York	896	77.2	62.8	38.1	38.3	65.6	916	94.0	85.0	58.5	39.1	83.0
North Carolina	303	39.9	30.4	21.8	19.1	51.5	483	59.6	42.9	23.8	20.9	26.3
North Dakota	31	67.7	38.7	25.8	19.4	74.2	59	81.4	42.4	15.3	16.9	18.6
Ohio	574	38.9	31.5	20.0	15.7	48.3	398	60.1	37.4	28.6	20.9	30.9
Oklahoma	148	75.0	68.2	38.5	40.5	77.7	204	81.9	68.6	23.5	19.6	68.6
Oregon	170	54.1	39.4	27.6	21.8	63.5	221	89.1	72.9	27.1	19.5	56.6
Pennsylvania	586	51.0	32.4	24.1	20.3	42.7	524	62.0	40.1	23.3	16.4	17.9
Puerto Rico	88	40.9	44.3	17.0	20.5	67.0	140	41.4	41.4	13.6	13.6	34.3
Rhode Island	62	62.9	50.0	22.6	21.0	35.5	52	78.8	57.7	42.3	36.5	26.9
South Carolina	121	33.1	33.9	29.8	23.1	44.6	113	72.6	48.7	22.1	15.0	34.5
South Dakota	48	47.9	33.3	18.8	22.9	45.8	62	87.1	40.3	27.4	24.2	35.5
Tennessee	292	51.4	28.8	26.0	17.5	41.1	226	50.4	31.9	23.5	24.3	22.1
Texas	361	58.4	46.3	43.8	30.7	53.2	484	70.2	55.4	24.0	16.5	34.3
Utah	116	51.7	57.8	25.0	26.7	70.7	233	68.7	62.7	33.5	30.9	48.5
Vermont	76	47.4	46.1	34.2	32.9	63.2	46	93.5	63.0	54.3	41.3	69.6
Virginia	273	52.4	33.3	23.1	17.9	45.8	226	64.2	41.2	23.5	21.7	28.3
Washington	283	54.4	30.0	15.2	12.7	46.3	425	78.6	49.9	15.3	11.3	33.9
West Virginia	113	33.6	27.4	22.1	15.9	40.7	106	50.9	36.8	32.1	24.5	25.5
Wisconsin	430	37.0	30.9	15.6	14.0	47.4	277	60.6	47.7	28.9	30.0	37.2
Wyoming	51	58.8	33.3	13.7	17.6	51.0	58	72.4	72.4	50.0	34.5	43.1

* Data from National Mental Health Services Survey, 2016.

^†^ Data from National Survey of Substance Abuse Treatment Services, 2016.

^§^ Does not include Commonwealth of the Northern Mariana Islands, Federated States of Micronesia, Guam, Republic of Palau, or U.S. Virgin Islands.

By state, the percentage of facilities offering tobacco cessation counseling ranged from 20.5% (Idaho) to 68.2% (Oklahoma) among mental health facilities and from 26.9% (Kentucky) to 85.0% (New York) among substance abuse treatment facilities. The percentage of facilities with smoke-free campus policies ranged from 19.9% (Idaho) to 77.7% (Oklahoma) among mental health treatment facilities and from 10.0% (Idaho) to 83.0% (New York) among substance abuse treatment facilities. In 31 states, fewer than half of mental health facilities had smoke-free campuses (Figure 1), and fewer than half of substance abuse facilities had smoke-free campuses in 43 states, the District of Columbia, and Puerto Rico (Figure 2).

**FIGURE 1:**
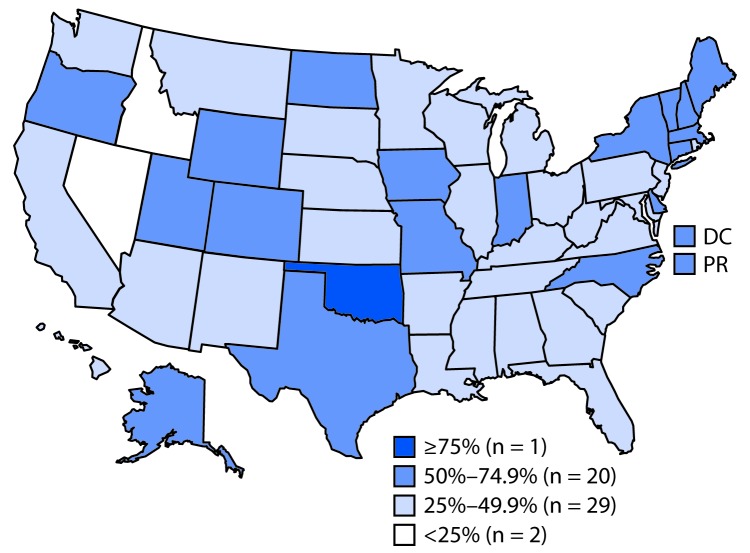
Percentage of mental health treatment facilities that prohibit smoking in all indoor and outdoor locations — National Mental Health Services Survey, United States, 2016 **Abbreviations:** DC = District of Columbia; PR = Puerto Rico.

**FIGURE 2:**
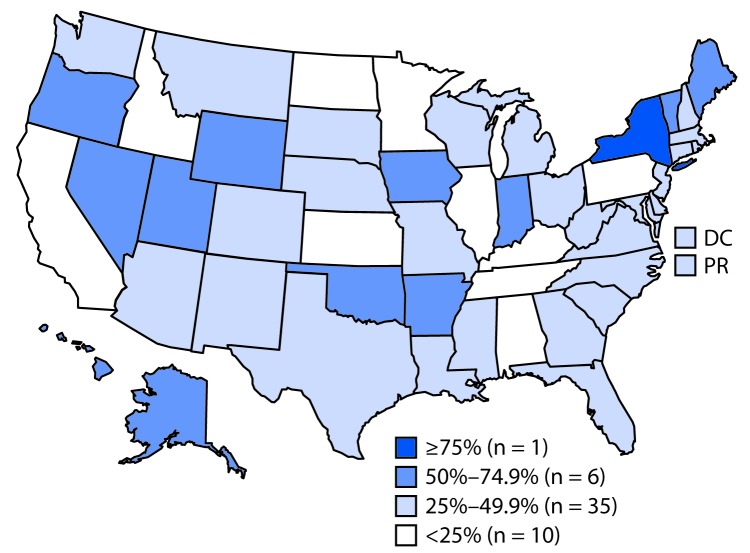
Percentage of substance abuse treatment facilities that prohibit smoking in all indoor and outdoor locations — National Survey of Substance Abuse Treatment Services, United States, 2016 **Abbreviations:** DC = District of Columbia; PR = Puerto Rico.

## Discussion

Opportunities exist to enhance both smoke-free environments and tobacco cessation treatment in mental health and substance abuse treatment settings. In 2016, fewer than half of such facilities in the United States (including Puerto Rico) offered evidence-based tobacco cessation treatments, and substantial proportions of facilities with smoke-free campus policies did not report offering tobacco cessation counseling or medications. Given that tobacco cessation in behavioral health treatment could improve both physical and behavioral health outcomes, and continued smoking worsens those outcomes, behavioral health treatment facilities are an important setting for evidence-based tobacco cessation interventions (*3*,*4*).

Several factors might contribute to the relatively low availability of evidence-based tobacco cessation treatments and smoke-free environments in behavioral health settings. First, some behavioral health treatment providers have viewed smoking cessation as a low priority relative to treatment of behavioral health conditions (*2*,*5*). Although smoking cessation could improve behavioral health outcomes (*3*,*4*), some providers are concerned that receiving smoking cessation treatment or quitting smoking during behavioral health treatment could exacerbate mental health symptoms or jeopardize substance abuse recovery (*3*,*4*). However, the latest evidence does not support these concerns (*1*,*3*,*5*). Notwithstanding, it is important to monitor patients during smoking cessation; for example, because smoking increases metabolism of some psychotropic medications, dosages might need to be adjusted among patients who have quit (*1*,*3*,*5*). Some behavioral health providers also believe that behavioral health patients who smoke are either unable or unwilling to quit (*1*–*3*). However, many smokers with behavioral health conditions want to quit smoking, are able to quit, and benefit from evidence-based smoking cessation treatments (*1*–*3*). Second, a lack of provider incentives for delivering tobacco cessation treatment, including reimbursement challenges, might pose additional barriers (*5*). Finally, in the past, the tobacco industry has opposed smoke-free psychiatric hospital policies, donated cigarettes to mental health facilities, and funded research suggesting that patients with psychiatric illnesses need tobacco for self-medication (*1*,*2*).

Several actions could help address actual and perceived barriers to integrating tobacco dependence treatment into behavioral health treatment. These actions could include removing administrative and financial barriers to delivery of cessation interventions and integrating tobacco screening and treatment protocols into facilities’ workflows and electronic health record systems (*1*,*2*,*5*). In addition, outreach to behavioral health providers could emphasize that their patients can benefit from evidence-based cessation treatments, although longer duration or more intensive cessation treatments might be indicated (*1*,*5*).

Progress has been achieved in recent years in addressing tobacco use in behavioral health treatment settings.^§^ For example, New York adopted regulations requiring tobacco-free campus policies in state-funded or state-certified substance abuse treatment programs and expanded Medicaid cessation benefits to allow unlimited quit attempts per year. Oklahoma improved access to treatment by eliminating copayments and prior authorization for tobacco cessation treatment for Medicaid enrollees. In addition, Oklahoma required that all substance abuse treatment facilities and state-contracted mental health treatment facilities implement tobacco-free campus policies, conduct evidence-based clinical cessation interventions, and document tobacco quitline referrals. In 2016, the Smoking Cessation Leadership Center and the American Cancer Society convened health experts, organizations, and federal agencies, including CDC and SAMHSA, to create a national action plan to reduce smoking among persons with behavioral health issues from 34% in 2015 to 30% by the year 2020.^¶^

The association between cigarette smoking and both substance abuse onset and relapse reinforces the importance of tobacco prevention and cessation efforts across the lifespan. Preventing tobacco use initiation might be viewed as a primary substance abuse prevention strategy because of the association between adolescent cigarette smoking and subsequent drug dependence (*6*). Animal models suggest that adolescent exposure to nicotine increases susceptibility to addiction to other substances (*6*), including alcohol, cocaine, methamphetamine (*6*), and opioids (*7*). In the current context of rising demand for opioid addiction treatment,** it is noteworthy that nicotine and opioid addictions are mutually reinforcing, whereas smoking cessation is associated with long-term abstinence after opioid treatment (*8*,*9*). In addition, cigarette smoking and chronic pain might interact in ways that might make smokers with chronic pain especially susceptible to opioid misuse (*8*). Therefore, efforts to increase tobacco cessation and prevent youth tobacco initiation, including during substance abuse treatment, are important components of a comprehensive strategy to prevent and reduce substance abuse.

The findings in this report are subject to at least three limitations. First, data are self-reported by facility personnel and might be subject to bias. Second, data are at the facility level rather than patient level and facilities are counted equally regardless of size, precluding estimates of individual patients’ access to cessation interventions. Finally, use of cessation treatments or implementation of smoke-free policies could not be assessed, including whether policies permit use of e-cigarettes and other tobacco products. Tobacco-free campus policies that prohibit all forms of tobacco product use, including use of e-cigarettes and smokeless tobacco, can support tobacco cessation, reinforce tobacco-free norms, and eliminate exposure to secondhand tobacco product emissions (*6*).

A comprehensive effort to reduce tobacco-related disparities among persons with behavioral health conditions includes clinical cessation interventions, as well as population-level measures to reduce the appeal, accessibility, and social acceptability of tobacco use outside the clinical context (*1*). Proven interventions, including raising tobacco prices, implementing comprehensive smoke-free laws, conducting media campaigns, and providing barrier-free access to proven cessation treatments, are critical to reduce smoking-related disease and death in the United States (*1*,*6*).

**Summary**

**What is already known about this topic?**

Many persons with mental or substance use disorders who smoke want to and can quit smoking.

**What is added by this report?**

In 2016, among mental health facilities, 49% screened patients for tobacco use, 38% offered tobacco cessation counseling, and 49% had smoke-free campuses; corresponding estimates for substance abuse facilities were 64%, 47%, and 35%, respectively. Approximately one in four behavioral health treatment facilities offered nicotine replacement therapy; one in five offered non-nicotine cessation medications.

**What are the implications for public health practice?**

Tobacco-free campus policies and integration of tobacco cessation interventions in behavioral health treatment facilities could decrease tobacco-related disease and death and could improve behavioral health outcomes among persons with mental and substance use disorders.

## Footnotes

* N-MHSS: https://www.samhsa.gov/data/sites/default/files/2016_National_Mental_Health_Services_Survey.pdf. N-SATSS: https://www.samhsa.gov/data/sites/default/files/2016_NSSATS.pdf. Excluded for N-MHSS were facilities whose client counts were included in other facilities’ counts and whose facility characteristics were not reported separately and facilities that provided administrative services only. Excluded for N-SSATS were nontreatment halfway houses, solo practices not approved by the state agency for inclusion, and facilities that treated incarcerated clients only.

† This report does not include data collected from the Commonwealth of the Northern Mariana Islands, the Federated States of Micronesia, Guam, the Republic of Palau, or the U.S. Virgin Islands because they are not reported separately by N-MHSS and N-SSATS.

§ https://smokingcessationleadership.ucsf.edu/leadership-and-policy-academies.

¶ https://smokingcessationleadership.ucsf.edu/national-partnership-behavioral-health-and-tobacco-use.

** See example at https://psychcentral.com/news/2016/12/26/many-rural-opioid-users-on-long-waiting-lists-for-treatment/114354.html.
